# The plasmid-encoded transcription factor ArdK contributes to the repression of the IMP-6 metallo-β-lactamase gene *bla*_IMP-6_, leading to a carbapenem-susceptible phenotype in the *bla*_IMP-6_-positive *Escherichia coli* strain A56-1S

**DOI:** 10.1371/journal.pone.0208976

**Published:** 2018-12-11

**Authors:** Takaya Segawa, Tsuyoshi Sekizuka, Satowa Suzuki, Keigo Shibayama, Mari Matsui, Makoto Kuroda

**Affiliations:** 1 Pathogen Genomics Center, National Institute of Infectious Diseases, Shinjuku-ku, Tokyo, Japan; 2 Antimicrobial Resistance Research Center, National Institute of Infectious Diseases, Higashimurayama, Tokyo, Japan; 3 Bacteriology II, National Institute of Infectious Diseases, Musashimurayama, Tokyo, Japan; Cornell University, UNITED STATES

## Abstract

Carbapenemase-producing Enterobacteriaceae (CPE) are a global concern because these bacteria are resistant to almost all β-lactams. Horizontal interspecies gene transfer via plasmid conjugation has increased the global dissemination of CPE. Recently, an Enterobacteriaceae strain positive for carbapenemase gene but showing a carbapenem-susceptible phenotype was identified, suggesting that these susceptible strains may be challenging to detect solely via antimicrobial susceptibility tests without molecular analysis. Here, we isolated a *bla*_IMP-6_ carbapenemase-gene positive but imipenem- and meropenem-susceptible *Escherichia coli* (ISMS-E) strain A56-1S (imipenem and meropenem minimum inhibitory concentration, ≤ 0.125 mg/L), from a human urine specimen in Japan. A56-1S was carbapenemase negative by the Carba NP test, suggesting that IMP-6 production was low or undetectable. Thus, to characterize the mechanism of this phenotype, a meropenem-resistant *E*. *coli* A56-1R strain was obtained using meropenem-selection. A56-1R was positive for carbapenemase production by the Carba NP test, and *bla*_IMP-6_ transcription in A56-1R was 53-fold higher than in A56-1S, indicating that *bla*_IMP-6_ in A56-1S is negatively regulated at the transcriptional level. Comparative genomic analysis between the two strains revealed that the alleviation of restriction of DNA (*ardK*) gene encoding a putative transcription factor is disrupted by the IS*26* insertion in A56-1R. A cotransformation assay of *ardK* and the regulatory element upstream of *bla*_IMP-6_ showed repression of *bla*_IMP-6_ transcription, indicating that ArdK negatively modulates *bla*_IMP-6_ transcription. ArdK binding and affinity assays demonstrated that ArdK directly binds to the regulatory element upstream of *bla*_IMP-6_ with dissociation constant values comparable to those of general transcription factors. The IMP-6 carbapenemase showed low hydrolytic activity against imipenem, resulting in an imipenem-susceptible and meropenem-resistant (ISMR) phenotype (previously reported as a stealth phenotype). However, the low expression of IMP-6 in the A56-1S strain could be a typical characteristic of ISMS-E due to gene repression, indicating that conventional antimicrobial susceptibility tests might be unable to detect such strains even when using both imipenem and meropenem. Bacteria that exhibit the ISMS phenotype could play a potential role as undetectable reservoirs and might facilitate gene transfer to other organisms while avoiding detection.

## Introduction

The spread of carbapenem-resistant Enterobacteriaceae (CRE) is a global concern, because carbapenem is considered the last resort for treating infectious diseases caused by gram-negative bacteria, such as Enterobacteriaceae. Carbapenemase-producing Enterobacteriaceae (CPE) are resistant to almost all β-lactams, and the plasmid-borne carbapenemase genes can be transferred to other bacteria by conjugation [1–3].

To date, meropenem (MEM) susceptibility testing remains the most efficient method for screening CPE, and provides the best balance between sensitivity and specificity [4]. The minimum inhibitory concentration (MIC) breakpoint of Enterobacteriaceae for MEM is defined under the European Committee on Antimicrobial Susceptibility Testing (EUCAST) guidelines as, susceptible for ≤ 2 mg/L and resistant for > 8 mg/L [5]. For CPE detection, MIC breakpoints do not always exhibit good sensitivity. Therefore, the EUCAST recommends the detection of potential CPE with lower screening cut-off values (e.g., MEM MIC > 0.125 mg/L) for further investigation [6].

In 2010, we collected clinical isolates of Enterobacteriaceae resistant to fluoroquinolone, aminoglycoside, and either carbapenem (IPM or MEM MIC ≥ 8 mg/L) or ceftazidime (MIC ≥ 32 mg/L), from hospitals in Japan to investigate the dissemination of carbapenemase producers. In this survey, ceftazidime resistance was also investigated to identify carbapenemase producers with imipenem (IPM) MICs below the breakpoint [7]. In total, we collected 153 potential CPE, on which PCR-based investigation (*bla*_NDM-1_, *bla*_KPC_, *bla*_IMP_, *bla*_VIM-2_, *bla*_OXA-48-like_) was performed [7]. Among these clinical isolates, we identified one *Escherichia coli* isolate (designated A56-1S) carrying *bla*_IMP-6_ but exhibiting an IPM-susceptible MEM-susceptible (ISMS) phenotype (IPM and MEM MICs ≤ 0.125 mg/L, which is below the screening cut-off value for CPE) and negative for carbapenemase production by the Carba NP test. Based on these phenotypes, A56-1S was not recognized as an IMP-6 producer.

Compared to IMP-1 metallo-β-lactamase (MBL), IMP-6 MBL is an IMP variant with a S214G amino acid substitution in the catalytic domain. As a result, IMP-6 exhibits significantly weakened enzymatic activity towards IPM but not MEM [8] and IMP-6 producers of the Enterobacteriaceae generally exhibit an IPM-susceptible MEM-resistant (ISMR) phenotype upon MIC testing [7,9,10]. Therefore, MEM is recommended for the detection of IMP-6 producers. The Carba NP test is also recommended to improve the detection of IMP-6 producers because of its high sensitivity. However, we identified the Carba NP test-negative *bla*_IMP-6_-positive *E*. *coli* A56-1S strain. We therefore speculated that IMP-6 expression in A56-1S could be significantly repressed, resulting in an ISMS phenotype.

Here, we performed comparative genomic as well as transcriptional analysis, and carbapenemase-production assays to demonstrate that the mechanism behind ISMS phenotype is involved to all phenotypes associated with carbapenem susceptibility.

## Materials and methods

### Bacterial strains

The ISMS *bla*_IMP-6_-harboring *E*. *coli* strain A56-1S was isolated from a urine specimen of a patient in Japan (Table 1). The MEM-resistant *E*. *coli* A56-1R strain was selected from a A56-1S culture grown at 35°C for 18 h on Luria-Bertani (LB) agar supplemented with 1 mg/L MEM as a selection agent. A single colony was then cultured on fresh LB agar with MEM. The *bla*_IMP-6_ sequence was determined using PCR and direct sequencing as previously reported [7].

**Table 1:** Bacterial strains and plasmids used in this study.

Strain or plasmid		Description		Reference or source
**Strains (*E*. *coli*)**				
	**A56-1S**	**Clinical isolate harboring *bla***_**IMP-6**_ **from a patient urine sample. Imipenem and meropenem susceptible phenotype.**		**This study**
	**A56-1R**	**Strain selected from A56-1S with MEM selection. Imipenem susceptible but meropenem resistant phenotype.**		**This study**
	**OmniMAX**	***E*. *coli* strain for cloning assay.**		**Thermo Fisher Scientific**
	**Omni-UPS**	**OmniMAX transfected with pACYC184-UPS.**		
	**Omni-UPS_A**	**OmniMAX transfected with pACYC184-UPS and pCR-XL-2- *ardK*.**		**This study**
	**Omni-UPS_mA**	**OmniMAX transfected with pACYC184-UPS and pCR-XL-2- m*ardK*.**		**This study**
	**Omni-UPR**	**OmniMAX transfected with pACYC184-UPR.**		
	**Omni-UPR_A**	**OmniMAX transfected with pACYC184-UPR and pCR-XL-2-*ardK*.**		**This study**
	**Omni-UPR_mA**	**OmniMAX transfected with pACYC184-UPR and pCR-XL-2-m*ardK*.**		**This study**
**Plasmids**				
	**pA56-1S**	***bla***_**CTX-M-2**_**, *bla***_**IMP-6**_**, *sul1*, *aadA2*, *tetA*** **53-kbp IncN wild-type plasmid in A56-1S.**		**This study (GenBank ID: AP018362)**
	**pA56-1R**	***bla***_**CTX-M-2**_**, *bla***_**IMP-6**_**, *sul1*, *aadA2*, *tetA*** **53-kbp IncN plasmid in A56-1R.**		**This study (GenBank ID: AP018363)**
	**pCR-XL-2-TOPO**	**Vector for cloning *ardK*.** **Selectable marker, Ampicillin and Kanamycin resistance genes.**		**Thermo Fisher Scientific**
	**pCR-XL-2-*ardK***	**pCR-XL-2-TOPO carrying *ardK* in multiple cloning site**		**This study**
	**pCR-XL-2-m*ardK***	**pCR-XL-2-TOPO carrying mutated *ardK* in multiple cloning site**		**This study**
	**pACYC184**	**Vector for cloning region upstream of *bla***_**IMP-6**_. **Selectable marker, Chloramphenicol and Tetracycline resistance genes.**		**Nippon Gene**
	**pACYC184-UPS**	**pACYC184 carrying region upstream of *bla***_**IMP-6**_ **of A56-1S behind *tetC***		**This study**
	**pACYC184-UPR**	**pACYC184 carrying region upstream of *bla***_**IMP-6**_ **of A56-1R behind *tetC***		**This study**
	**pET SUMO**	**Vector for expression of His-tagged protein.** **Selectable marker, Kanamycin resistance gene.**		**Thermo Fisher Scientific**

### Antimicrobial susceptibility testing and CPE screening

Antimicrobial susceptibility to ceftazidime (CAZ), cefmetazole (CMZ), cefotaxime (CTX), amikacin (AMK), gentamicin (GEN), ciprofloxacin (CIP), fosfomycin (FOF), and trimethoprim-sulfamethoxazole (SXT) was tested with a Vitek2 system (Sysmex bioMérieux Co., Ltd., Tokyo, Japan). The agar dilution method was used to determine susceptibility to IPM and MEM. Antimicrobial breakpoints were determined using guidelines in EUCAST Clinical breakpoints-bacteria (v 8.1) [5]. Carbapenemase production by both strains was determined using the Carba NP test, as described previously [11].

### Measurement of carbapenemase gene transcript by quantitative reverse transcription PCR (qRT-PCR)

To analyze IMP-6 MBL production, the transcript levels of *bla*_IMP-6_ was measured by qRT-PCR. Total bacterial RNA was prepared from bacteria grown in 5 mL of LB broth to an optical density of 0.2–0.3 (OD_600_) measured using a GENESYS 20 visible spectrophotometer (Thermo Fisher Scientific, Waltham, MA, USA). One mL of the bacterial culture was centrifuged at 10,000 × *g* for 1 min at 20°C to collect the bacterial pellet, which was resuspended in 300 μL TE buffer [1 mM Tris-HCl (pH 7.8), 0.1 mM EDTA]. Then, 30 μL of 10% sodium dodecyl sulfate (SDS) and 330 μL of acid phenol (Wako, Osaka, Japan) were added to the suspension and mixed using a vortex mixer for 1 min. This mixture was then centrifuged at 15,000 × *g* for 10 min at 20°C. The supernatant was collected, and total RNA was purified using the RecoverAll Total Nucleic Acid Isolation Kit for FFPE (Thermo Fisher Scientific) following the manufacturer’s instructions. The total RNA concentration was measured by a Qubit 2.0 fluorometer and the Qubit RNA BR Assay Kit (Thermo Fisher Scientific). qRT-PCR was performed with the ReverTra Ace qPCR RT Kit and KOD SYBR qPCR Mix (Toyobo, Osaka, Japan) using qPCR primers (Table 2). The cycling conditions were as follows, 98°C for 2 min, followed by 40 cycles at 98°C for 10 s, 58°C for 10 s, and 68°C for 30 s. Fluorescence intensities were measured using a QuantStudio 3 real-time PCR system (Thermo Fisher Scientific). The results were normalized using expression of the RNA polymerase β-subunit (*rpoB*) gene as internal control, as previously reported [7].

**Table 2:** Oligonucleotides used in this study.

Name	Sequence (5'-3')	Reference or source
**IMP-6_qPCR_F**	**GTTTTGCAGCATTGCTACCG**	[7]
**IMP-6_qPCR_R**	**CCCCACCCGTTAACTTCTTC**	[7]
**rpoB_qPCR_F**	**GAGTTCTTCGGTTCCAGCCA**	[7]
**rpoB_qPCR_R**	**GAGTGCGGAGATACGACGTT**	[7]
**IntI1_pACYC184_F**	**GAGTGGAACCAACCGGTAACAGCCTTTCTGGCTG**	**This study**
**IntI1_pACYC184_R**	**GTATCGTGGTATCACTTAGTTGCTTGGTTTTGATGG**	**This study**
**pACYC184_InFusion_F**	**GTGATACCACGATACTATGAC**	**This study**
**pACYC184_InFusion_R**	**CGGTTGGTTCCACTCTCTGTTG**	**This study**
***ardK*_cloning_F**	**GAGTGGAACCAACCGCTGCTGCCGCACCGGGCC**	**This study**
***ardK*_cloning_R**	**GTATCGTGGTATCACTTATTTTTTTTCCATTTCCGAG**	**This study**
***ardK*_TA_F**	**ATGGCTCAGAAAAACAGAAT**	**This study**
***ardK*_TA_R**	**TTATTTTTTTTCCATTTCCGAGGTCG**	**This study**
**ArdK_EMSA_region_F_5_biotin**	**AGGAAATACGCGGTCGTTAC**	**This study**
**ArdK_EMSA_region_R**	**GAATAAGACAAAAGGCTGCC**	**This study**
**ArdK_EMSA_region_1_F**	**AGGAAATACGCGGTCGTTACCCGCTCAGGT**	**This study**
**ArdK_EMSA_region_5_R**	**GAATAAGACAAAAGGCTGCCTCATCGCTAA**	**This study**

### Preparation of chromosomal and plasmid DNA for next-generation sequencing (NGS)

To separate plasmids from chromosomal DNA, S1 nuclease pulsed-field gel electrophoresis (PFGE) was performed according to Barton et al. with modifications [12]. The total DNA in agarose gel plugs was incubated in distilled water for 30 min at 20°C. Then, the plugs were incubated with 30 U of S1 nuclease (TaKaRa, Shiga, Japan) for 30 min at 4°C, followed by incubation for 40 min at 37°C. Reactions were stopped by incubation for 5 min at 20°C with 0.5 M EDTA. The plugs were rinsed using 10 mM Tris-HCl/1 mM EDTA (pH 8.0) and incubated for 1 h at 4°C with 0.5 × TBE buffer (50 mM Tris, 45 mM boric acid, and 0.5 mM EDTA). The total DNA digested with S1 nuclease in plugs was loaded onto a 1% agarose gel and run with a CHEF Mapper XA pulsed-field electrophoresis system (Bio-Rad, Hercules, CA, USA) at 14°C in 0.5 × TBE buffer for 20 h under the following conditions: auto algorithm, 20-kb low molecular weight (MW), 250-kb high MW, and 6.0 V/cm. After electrophoresis, the agarose gel was stained with SYBR Gold nucleic acid gel stain (1/10,000 dilution; Thermo Fisher Scientific) for 1 h at 20°C. Bands were visualized using a WSE-5200A Printgraph 2M system (ATTO, Tokyo, Japan). The visualized bands were collected and stored at −30°C until whole-genome analysis was performed.

### Whole-genome analysis

Chromosomal and plasmid DNA were purified with the Zymoclean Gel DNA Recovery Kit (Zymo Research, Irvine, CA, USA), which was followed by DNA-Seq library preparation using the Nextera XT DNA Sample Preparation Kit (Illumina, San Diego, CA, USA), as previously described [13]. DNA libraries were sequenced on a MiSeq system (Illumina) in accordance with the manufacturer’s instructions (2 × 300-mer, paired end).

For PacBio long-read sequencing, long DNA fragments were prepared by extracting the bacterial cell pellet from a 20-ml overnight culture in SDS-phenol/chloroform with bead-beating for 10 min in ZR BashingBead Lysis Tubes (Zymo Research). After centrifugation at 10,000 × g for 5 min, the upper phase was subjected to electrophoresis on a 1% TAE (40 mM Tris-acetate, 1 mM EDTA) conventional agarose gel. The agarose gel was stained with fluorescent DNA-intercalating dye (GelRed; Biotium, Fremont, CA, USA), bands within the targeted DNA size range (15–40 kb) were excised under a Safe Imager blue light transilluminator (Thermo Fisher Scientific) to avoid DNA damage, and the DNA was purified using the Zymoclean Large Fragment DNA Recovery Kit (Zymo Research). Purified DNA was used to prepare a SMRTbell library (PacBio, Menlo Park, CA, USA). Single molecule real time (SMRT) sequencing was performed for one library on one SMRT cell with P6C4 chemistry on a PacBio RSII sequencer. To obtain the whole-genome sequence in circular form, the raw polymerase reads were analyzed using the HGAP v3.0 pipeline based on Celera *de novo* assembler and Quiver polishing script [14].

The class 1 integron has been classified as In1568 (*intI1*, *dfrA14*Δ3::IS*26*, Δ*aacA4*, *bla*_IMP-6_, *aadA2*, and *gcu52*) and In1569 ((*intI1*, *dfrA14*Δ3::IS*26*) in the INTEGRALL database (http://integrall.bio.ua.pt/) [15].

### Plasmid construction

DNA fragments of the intact and mutated *ardK* genes were amplified from pA56-1S (GenBank ID: AP018362) and pA56-1R (GenBank ID: AP018363) genomic DNA, respectively, using primers *ardK*_cloning_F and R (Table 2). The *ardK* PCR products were cloned with pCR-XL-2-TOPO (Thermo Fisher Scientific) following the manufacturer’s instructions.

DNA fragments from the region upstream of *bla*_IMP-6_ were amplified from pA56-1S (UPS; 3,524 bp; 3,193–6,716 nt in GenBank ID: AP018362) genomic DNA using IntI1_pACYC184_F and R, and from pA56-1R (UPR; 2,246 bp; 51,305–53,550 nt in GenBank ID: AP018363) genomic DNA using *ardK*_cloning_F and IntI1_pACYC184_R. The pACYC184 vector (Nippon Gene Co., Ltd., Tokyo, Japan) was linearized by PCR using the primer pair pACYC184_InFusion_F and R. PCR was performed with KOD FX Neo (Toyobo), and the cycling conditions were 95°C for 2 min; 40 cycles of 98°C for 10 s, 60°C for 30 s, and 68°C for 2 min, and a final extension at 68°C for 5 min. The PCR products from the region upstream of *bla*_IMP-6_ were inserted into a linearized pACYC184 vector using the In-Fusion HD Cloning Kit (TaKaRa). The cloned plasmids were propagated in One Shot OmniMAX 2 T1 Chemically Competent *E*. *coli* (Thermo Fisher Scientific) and purified using the PureYield Plasmid Miniprep System (Promega, Madison, WI, USA).

These plasmids were transformed into OmniMAX bacteria, and transformants carrying both UPS/UPR and intact/mutated *ardK* were selected using chloramphenicol and kanamycin. Some transformants were isolated and subjected to MEM susceptibility testing using broth microdilution, and carbapenemase production using the Carba NP test.

### Purification of recombinant protein

To prepare recombinant intact ArdK and mutated ArdK (mArdK) using the Champion pET SUMO Protein Expression System (Thermo Fisher Scientific), DNA fragments were amplified from A56-1S and A56-1R genomic DNA using primers *ardK*_TA_F and R (Table 2). PCR was performed using Ex Taq DNA Polymerase (TaKaRa), amplicons cloned into linearized pET SUMO and transformed into One Shot Mach1-T1 chemically competent *E*. *coli* (Thermo Fisher Scientific) in accordance with the manufacturer’s instructions. The cloned plasmid was purified using the PureYield Plasmid Miniprep System (Promega) and transformed into BL21 (DE3) *E*. *coli*. Cultures were grown in 50 mL of LB broth containing 50 mg/L kanamycin to an OD_600_ of 0.5, and protein expression was induced with 1 mM isopropyl-β-D-thiogalactopyranoside (IPTG), which was followed by incubation at 37°C for 6 h. The His6-SUMO-tagged recombinant proteins were purified under native conditions with TALON spin columns (TaKaRa) following the manufacturer’s instructions.

### Electrophoretic mobility shift assay (EMSA)

The ArdK-binding region on the plasmid was identified by EMSA. Biotin-labeled and unlabeled 107 bp DNA probes upstream of *bla*_IMP-6_ (pA56-1S, 4,760–4,866 nt) were amplified using primers (Table 2) ArdK_EMSA_region_F_5_biotin and ArdK_EMSA_region_R for biotin-labeled probe and ArdK_EMSA_region_1_F and ArdK_EMSA_region_5_R for unlabeled probe. EMSA was performed using the LightShift Chemiluminescent EMSA Kit (Thermo Fisher Scientific) following the manufacturer’s instructions with modifications. Then, 3 fmol of the biotin-labeled probes was incubated with 30 pmol of purified protein in a solution containing 20 mM Tris (pH 7.5), 100 mM KCl, 2 mM DTT, 10 mM EDTA, and 50 mg/L poly(dI-dC) at 20°C for 20 min. For the competition binding assay, 3 ng of double-stranded DNA purified from mammalian cells (Vero cells) was included in the binding reactions. The reactions were loaded on a 5% polyacrylamide TBE gel and electrophoresed at 100 V for 60 min at 4°C. Bands were detected using the Fusion SL4 imaging system (Vilber Lourmat, Marne-la-Vallée, France).

### Binding assay using biolayer interferometry

The interaction between ArdK and the 107 bp DNA probe upstream of *bla*_IMP-6_ (pA56-1S; 4,760–4,866 nt) was analyzed by biolayer interferometry using the Octet RED96 system (Pall Corp. FortéBIO, Fremont, CA, USA). The assays were performed using 200 μL of 1 × kinetics buffer (Pall Corp. FortéBIO) per well according to manufacturer’s instructions. A baseline was established in the buffer for 1 min, and the biotin-labeled probes (10 pmol) were loaded onto streptavidin sensors (Pall Corp. FortéBIO) for 5 min. The sensors were washed for 1 min and exposed to recombinant ArdK at different concentrations (0.2–0.8 nM) for 5 min (association step), which was followed by a dissociation step for 5 min. The binding kinetics were analyzed using Octet data acquisition software v 9.0 (Pall Corp. FortéBIO). The experiments were performed in duplicate.

### Nucleotide sequence accession numbers

The complete, annotated plasmid sequences of pA56-1S and pA56-1R were deposited in a public database of DNA Data Bank of Japan (accession numbers: pA56-1S, AP018362; pA56-1R, AP018363). The short- and long-read sequences of DNA-Seq were deposited in the DNA Data Bank of Japan (BioProject PRJDB6276, BioSample SSUB008334, DRA accession DRA006165).

## Results

### Differences in antimicrobial susceptibility patterns between A56-1S and A56-1R

We selected the MEM-resistant *E*. *coli* strain A56-1R from A56-1S by culturing on LB agar supplemented with 1 mg/L MEM (Table 1). The antimicrobial susceptibility patterns of A56-1S and A56-1R were tested using the agar dilution method and a Vitek2 instrument (Table 3). A56-1S was resistant to CAZ, CTX, GEN, CIP and SXT, and exhibited high or intermediate susceptibility to IPM, MEM, AMK and FOF (Table 3). The antimicrobial susceptibility pattern of A56-1R was similar to that of A56-1S with the exception of its response to CAZ, IPM and MEM (Table 3). For example, the MEM MIC of A56-1R was over 256-fold higher than that of A56-1S. Both A56-1S and A56-1R tested positive for *bla*_IMP-6_ and *bla*_CTX-M_ via PCR amplification.

**Table 3:** Antimicrobial susceptibility profiles of A56-1S and A56-1R strains.

	Minimum inhibitory concentration (mg/L)			
Strain	A56-1S	A56-1R	MIC breakpoint^*C*^	
			S	R
**IPM**^***a***^	**0.06**	**0.25**	**≤ 2**	**> 8**
**MEM**^***a***^	**0.125**	**32**	**≤ 2**	**> 8**
**CAZ**^***b***^	**16**	**≥ 64**	**≤ 1**	**> 4**
**CMZ**^***b***^	**32**	**≥ 64**	**-**	**-**
**CTX**^***b***^	**≥ 64**	**≥ 64**	**≤ 1**	**> 2**
**AMK**^***b***^	**≤ 2**	**≤ 2**	**≤ 8**	**> 16**
**GEN**^***b***^	**≥ 16**	**≥ 16**	**≤ 2**	**> 4**
**CIP**^***b***^	**≥ 4**	**≥ 4**	**≤ 0.25**	**> 0.5**
**FOF**^***b***^	**≤ 16**	**≤ 16**	**≤ 32**	**> 32**
**SXT**^***b***^	**≥ 320**	**≥ 320**	**≤ 2/38**	**> 4/76**

^*a*^Agar dilution method

^*b*^Vitek2

^*c*^EUCAST Clinical breakpoints-bacteria (v 8.1)

AMK, amikacin; CAZ, ceftazidime; CIP, ciprofloxacin; CMZ, cefmetazole; CTX, cefotaxime; FOF, fosfomycin; GEN, gentamicin; IPM, imipenem; MEM, meropenem; SXT, trimethoprim-sulfamethoxazole; S, susceptible; R, resistant; -, not listed.

### Differences in IMP-6 MBL production and *bla*_IMP-6_ transcript levels between A56-1S and A56-1R

**Fig 1:**
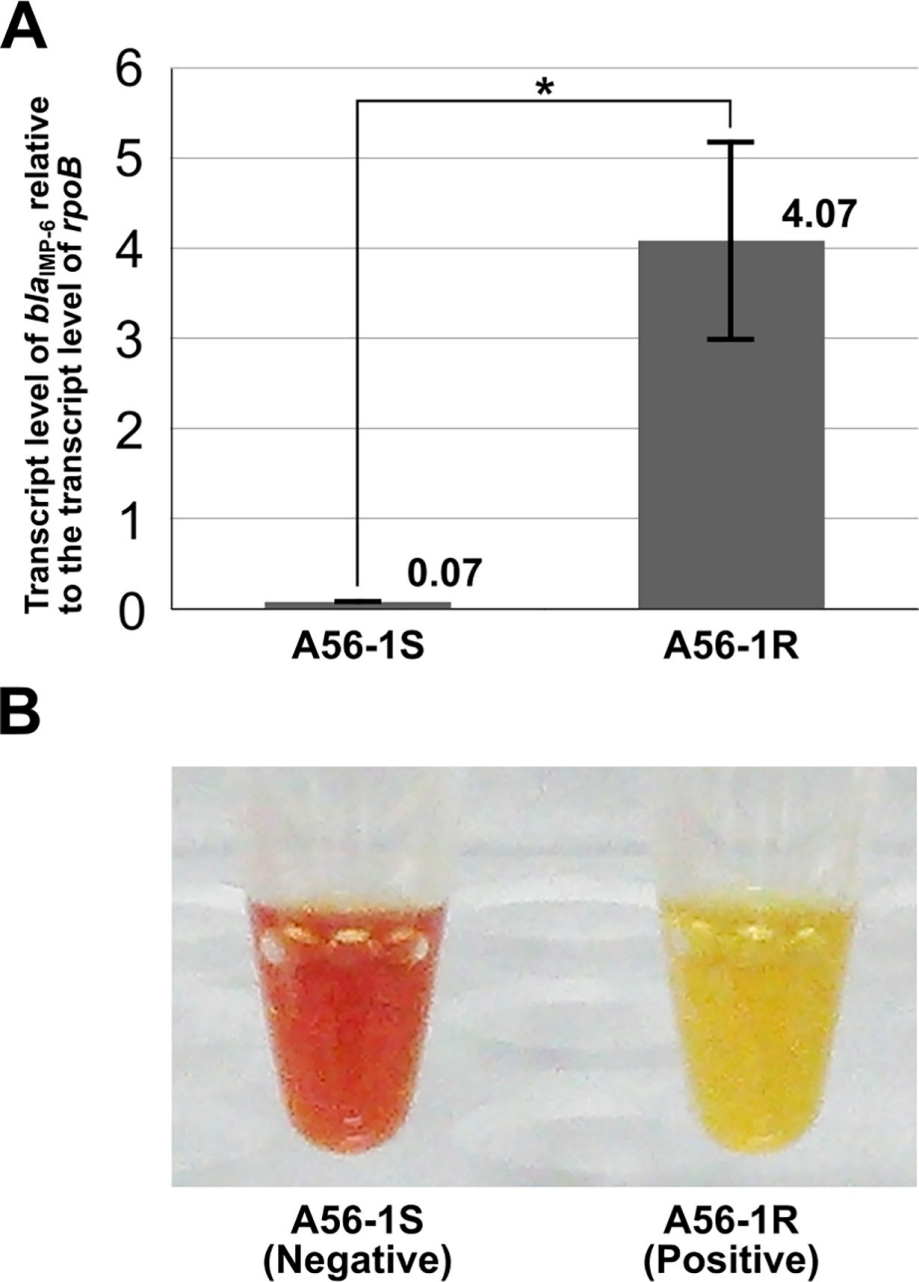
Differences in carbapenemase production and *bla*_IMP-6_ gene expression between A56-1S and A56-1R. For A56-1S and A56-1R, the carbapenemase production and *bla*_IMP-6_ transcript levels were analyzed using the Carba NP test and qRT-PCR, respectively. (A) The *bla*_IMP-6_ transcript levels were normalized to *rpoB* transcript levels. The graphs show the means of *bla*_IMP-6_ transcript level in each strain. The error bars show standard deviation of three replicates. The asterisk indicates a significant difference at *p* < 0.05 by Welch’s *t*-test. (B) In the Carba NP test, IPM hydrolysis by carbapenemase produces acid, leading to a change in color of phenol red from red (negative) to yellow (positive).

Differences in carbapenemase expression between A56-1S and A56-1R was tested by qRT-PCR of *bla*_IMP-6_ and the Carba NP test. The qRT-PCR analysis showed that the *bla*_IMP-6_ transcript level was 53-fold higher in A56-1R compared to A56-1S (Fig 1A). In the Carba NP test, A56-1S was negative and A56-1R was positive for carbapenemase production (Fig 1B). Therefore, carbapenemase production by these strains correlate with the observed meropenem susceptibility (Table 3).

### Comparative genome analysis between A56-1S and A56-1R

**Fig 2:**
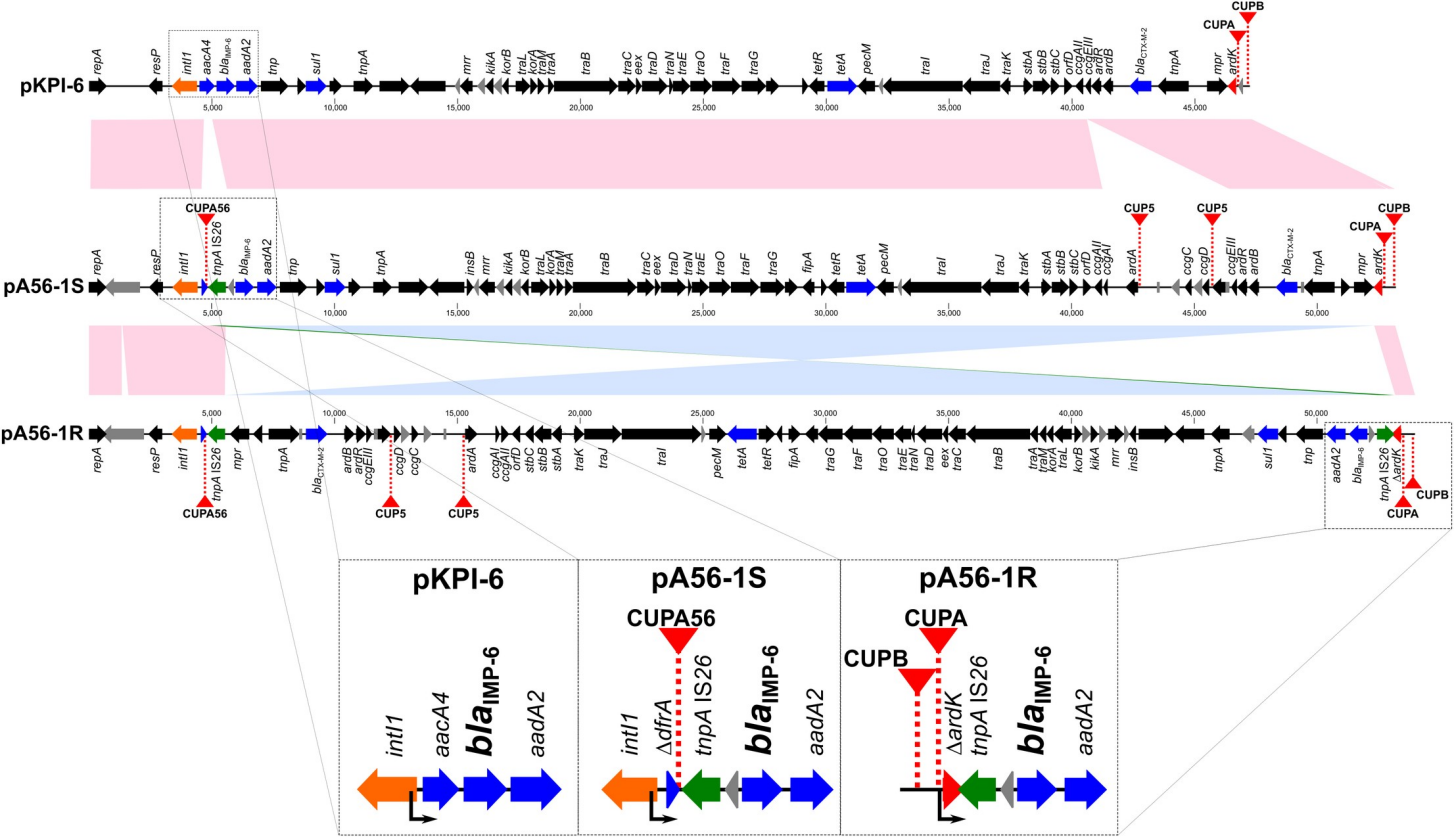
Schematic representation and pair-wise alignment of the *bla*_IMP-6_-positive plasmids pKPI-6, pA56-1S, and pA56-1R. The sequences of pA56-1S and pA56-1R were compared with the sequence of pKPI-6. The gene clusters of pA56-1S and pA56-1R were identical, with the exception of an additional IS*26* insertion in *ardK* on pA56-1R and subsequent inversion of the additional IS*26* sequence. The colored boxes indicate homologous regions with 100% nucleotide identity. Pink, the same orientation; blue, inversion; green, inverted insertion of an additional IS*26* element. Notable genes are represented by red, *ardK*; orange, integrase; green, IS*26*; blue, antimicrobial resistance genes; gray, hypothetical protein. Red arrowheads indicate conserved upstream (CUP) sequences which represent ArdK binding sites. Bent arrows indicate promoters upstream of *bla*_IMP-6_.

To analyze the difference in *bla*_IMP-6_ transcript levels between A56-1S and A56-1R, whole-genome sequencing was performed using NGS. The chromosomal and plasmid DNA were separated by S1 nuclease PFGE, and one chromosomal and three plasmid bands were observed for both strains (data not shown). We observed that the *bla*_IMP-6_-positive plasmids pA56-1S (GenBank ID: AP018362) and pA56-1R (GenBank ID: AP018363) were part of the IncN incompatibility group and carried other antimicrobial resistance genes (*bla*_CTX-M-2_, *sul1*, *aadA2*, *tetA*), a class 1 integron-integrase gene (*intI1*), and a conjugative transfer operon (Fig 2). Upstream of *bla*_IMP-6_ are located the promoters of *intI1* and *ardK* on pA56-1S and pA56-1R, respectively (Fig 2). A pairwise homology alignment showed that the proportion of homologous regions (at ≥ 99% nucleotide identity) between pA56-1S and pKPI-6 of *Klebsiella pneumoniae* KPI-6 strain (GenBank ID: AB616660 [9]) was 90.6% (Fig 2), suggesting that these plasmids share a common backbone.

There is a 791-bp difference in sequence length between pA56-1S (53,198 bp) and pA56-1R (53,989 bp) due to an additional IS*26* element that was transposed into the alleviation of restriction of DNA (*ardK*) coding sequence on pA56-1R, along with IS*26-*mediated inversion (Fig 2). This additional IS*26* element and the associated inversion caused disruption of the *ardK* gene, the product of which ArdK, is predicted to be a transcription factor belonging to the family of transcriptional repressor TrfB, as determined by its amino acid sequence using InterPro (similar to protein motif IPR032428). No sequence variations were identified in the chromosomal and other *bla*_IMP-6_-negative plasmid sequences between A56-1S and A56-1R (data not shown).

### Repression of IMP-6 MBL production by ArdK

**Fig 3:**
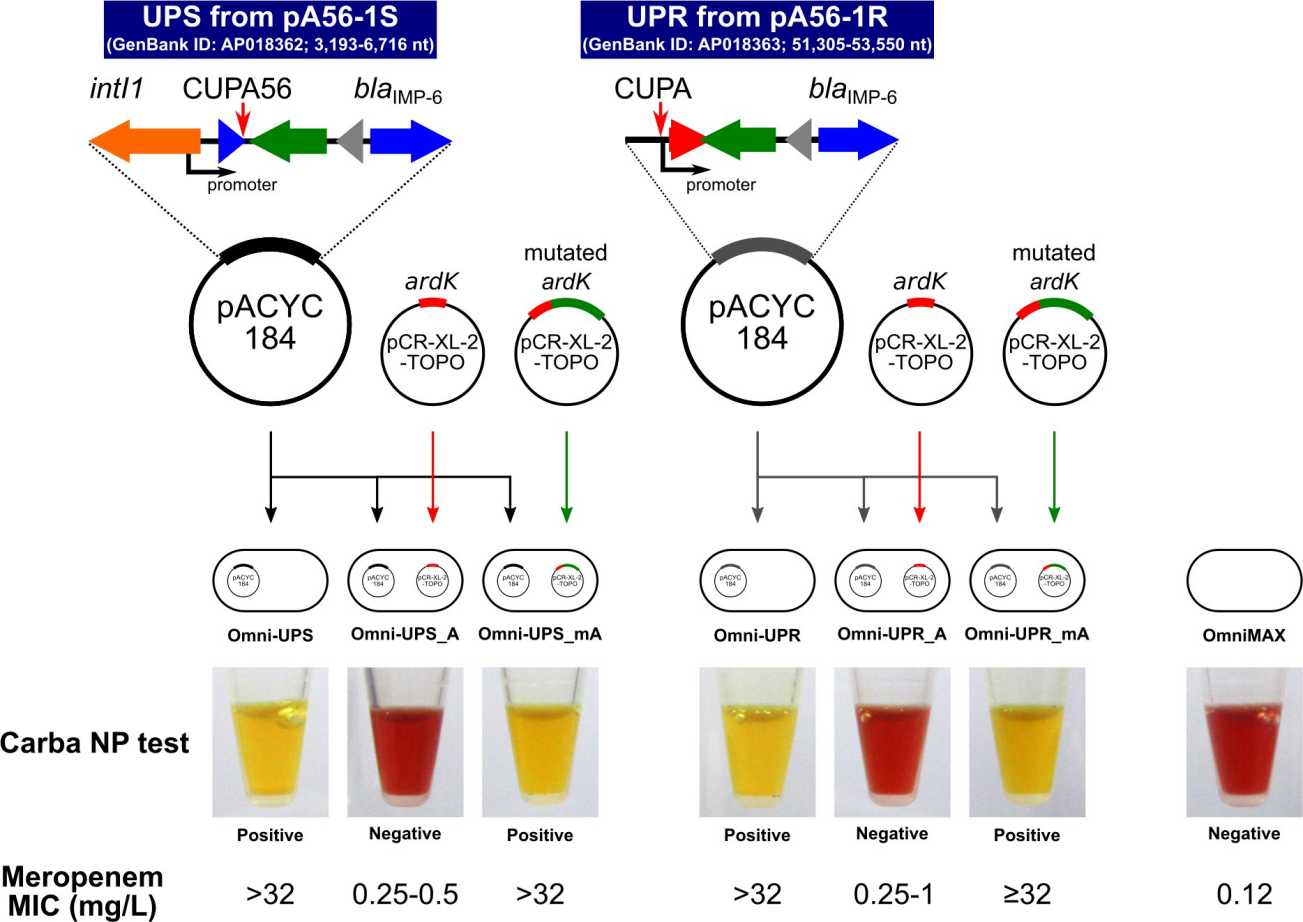
Analysis of repression of IMP-6 MBL production by ArdK using a reporter gene assay. To determine whether ArdK could repress IMP-6 MBL production, a *bla*_IMP-6_ reporter gene assay was performed using intact or mutated *ardK* gene. A schematic of the plasmid combinations is shown. Carbapenemase production and MEM susceptibility were tested by the Carba NP test and broth microdilution, respectively. In the Carba NP test, IPM hydrolysis by carbapenemase produces acid, leading to a change in color of phenol red from red (negative) to yellow (positive).

To determine whether ArdK mutated by IS*26* insertion is involved in *bla*_IMP-6_ expression, differences in IMP-6 MBL production between transformants with ArdK and mutated ArdK were analyzed using a *bla*_IMP-6_-based reporter assay. The *ardK* genes and regions upstream of *bla*_IMP-6_ from pA56-1S (UPS) and pA56-1R (UPR) were cloned separately into plasmids and transformed into an *E*. *coli* OmniMAX laboratory strain free from IncN-related genes including *ardK*, UPS and UPR on pA56-1S/pA56-1R (Fig 3). The transformants were tested using the Carba NP test and broth microdilution. The transformant carrying UPS (Omni-UPS) was positive on the Carba NP test and showed a MEM MIC of > 32 mg/L, suggesting that Omni-UPS produced IMP-6 MBL (Fig 3). The cotransformant with *ardK* (Omni-UPS_A) demonstrated negative results on the Carba NP test and MEM MIC of 0.25–0.5 mg/L, whereas the cotransformant with mutated *ardK* (Omni-UPS_mA) was positive on the Carba NP test and showed MEM MIC of > 32 mg/L (Fig 3), suggesting markedly reduced carbapenemase production in cotransformants carrying intact *ardK*. in addition, the Carba NP test and MEM MIC for UPR suggest that ArdK can modulate the expression of *bla*_IMP-6_ in transformants carrying either UPS or UPR (Fig 3).

### Characterization of the ArdK binding region on pA56-1S

**Fig 4:**
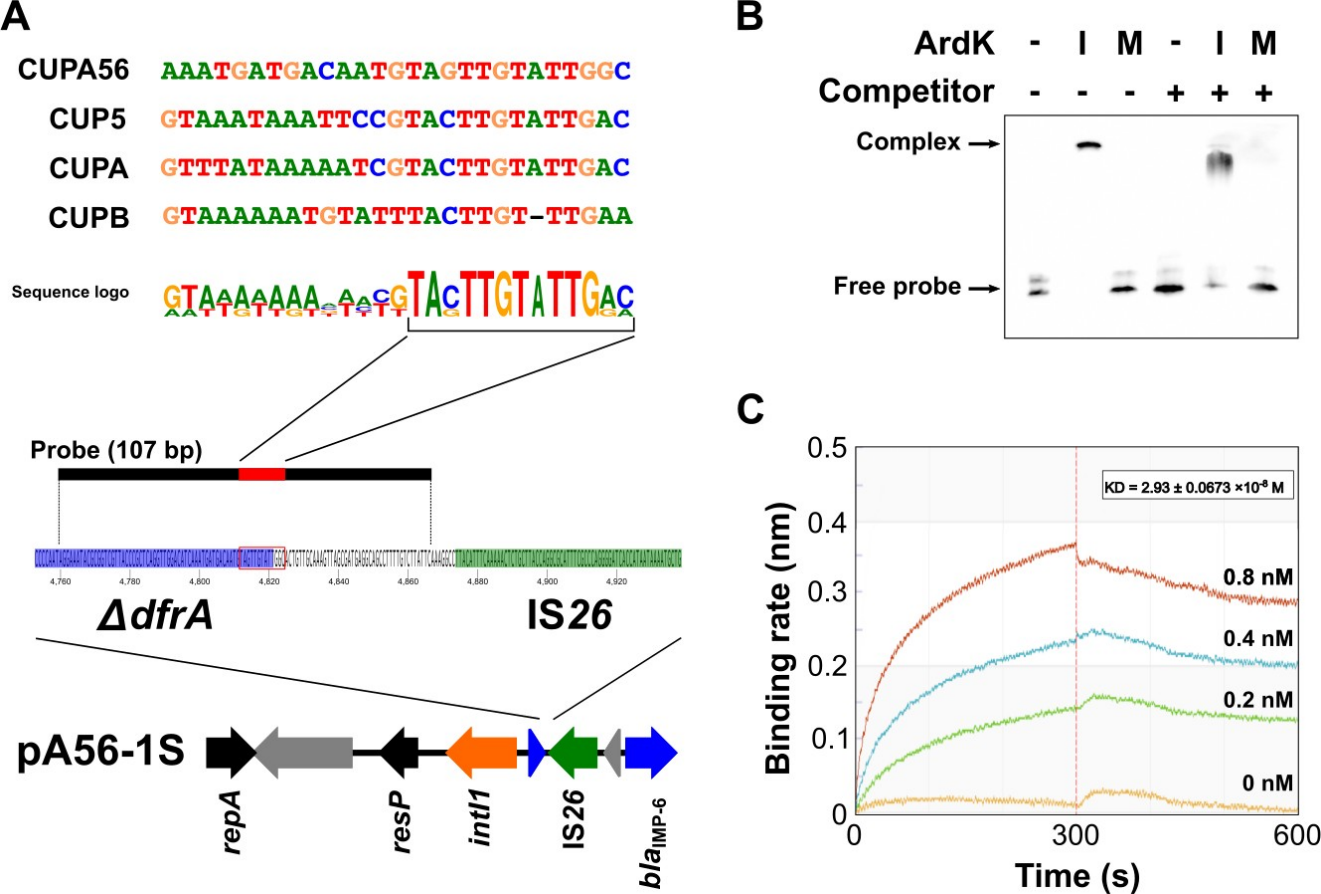
Characterization of the ArdK binding DNA sequence upstream of *bla*_IMP-6_ on pA56-1S by EMSA. The ArdK binding site was identified by EMSA. (A) The region upstream of *bla*_IMP-6_ on pA56-1S was compared with the ArdK binding sites of the CUP sequences, and the 13 consensus nucleotides (pA56-1S, 4812–4824 nt) were extracted from this region. (B) EMSA was performed with ArdK and the biotin-labeled 107 bp DNA probe (pA56-1S, 4760–4866 nt), which included the consensus sequence. For nonspecific competition assay, unlabeled mammalian genomic DNA was used as the nonspecific competitor. The arrows labeled complex and free probe correspond to bands of intact ArdK-DNA probe complex and DNA probe only, respectively. I, intact ArdK; M, mutated ArdK (mArdK); +, present; −, absent. (C) Determination of the DNA-binding affinity of ArdK. The binding affinity of ArdK to the biotin-labeled 107 bp DNA probe was measured using Octet. ArdK bound to the probe in 0–300 s (association step), and impurities were removed in 300–600 s (dissociation step). The colored lines indicate each ArdK concentration: orange, 0 nM; green, 0.2 nM; blue, 0.4 nM; red, 0.8 nM.

The above results suggest that ArdK could repress the transcription of *bla*_IMP-6_ (Fig 3). To identify the ArdK binding region upstream of *bla*_IMP-6_, EMSA was performed using a biotin-labeled DNA probe upstream of *bla*_IMP-6_ on pA56-1S. Three ArdK binding sites CUP5, CUPA and CUPB have been previously reported as conserved upstream sequences involved in the regulation of *ardB*, *ardK* and *repA*, respectively, on the IncN plasmid [16]. Comparative analysis of the upstream region of *bla*_IMP-6_ and these three sequences showed that the 13 consensus nucleotides (CUPA56, 4812–4824 nt in pA56-1S) were well aligned (CUP5, 84.6%; CUPB, 84.6%; CUPA, 69.2%; Fig 4A). A 107-bp DNA probe that included the consensus nucleotides labeled by biotin was amplified from pA56-1S, and EMSA was performed with the probe and recombinant ArdK (intact form on pA56-1S) or mArdK (C-terminus-deleted form on pA56-1R; Fig 4B). The results showed that the band corresponding to the probe was shifted by ArdK binding but not by mArdK. A nonspecific competition assay using a 30-fold weight excess of unlabeled mammalian genomic DNA showed that specific ArdK binding was retained as the band shift without competitor inhibition (Fig 4B). The binding affinity of ArdK for the 107 bp DNA probe was analyzed using the Octet RED96 system, and the equilibrium dissociation constant (*K*d) value was 2.93 ± 0.0673 × 10^−8^ M (Fig 4C).

## Discussion

**Fig 5:**
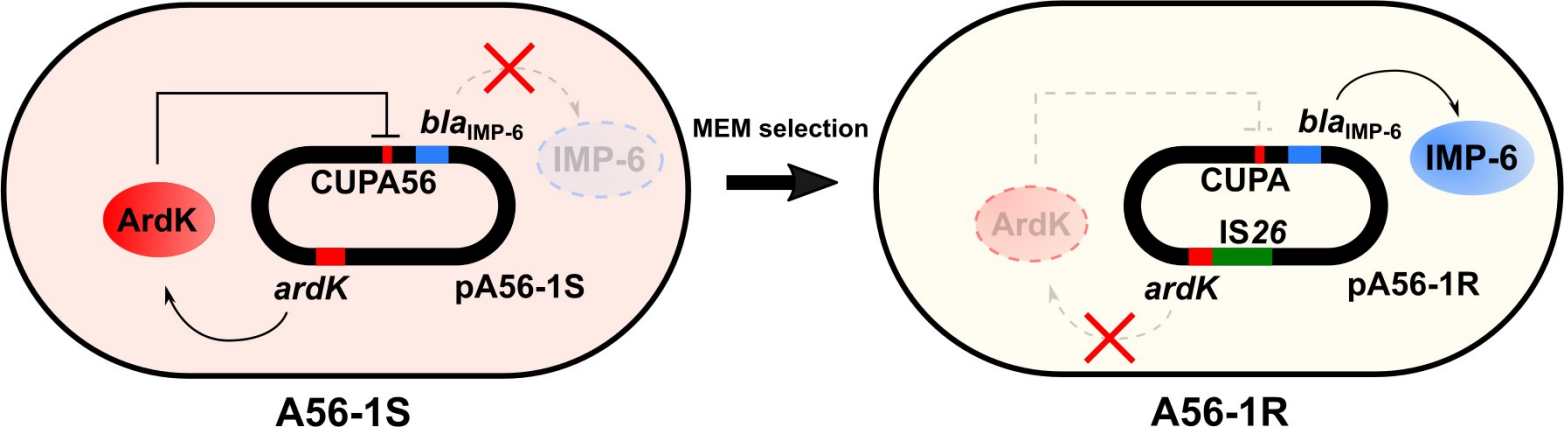
Schematic representation of the transcriptional regulation of *bla*_IMP-6_ by ArdK.

We investigated the mechanism of repression of carbapenemase production in *bla*_IMP-6_-positive ISMS *E*. *coli* A56-1S by comparative genomics with MEM-resistant A56-1R, revealing the presence of an *ardK* mutation by IS*26* insertion in pA56-1R (Figs 2, 3 and 5). The *ardK* gene is ubiquitously present on IncN plasmids and encodes the alleviation of restriction of DNA (ArdK) protein [16,17], which represses the transcription of genes such as *ardA* on the same plasmid [16]. ArdA contribute to overcoming the host restriction barrier during bacterial conjugation [18,19]. Moreover, ArdK can control the expression of replication protein RepA which affects the replication and copy number of the plasmid, suggesting that ArdK may contribute to establishing plasmid in a new host by controlling the expression of ArdA and RepA [16].

ArdK can also modulate its own expression by binding to the CUPA sequence that overlaps with the *ardK* promoter region and corresponds to the binding regions on pA56-1S (52,695–52,721 nt) and pA56-1R (53,486–53,512 nt) [16]. In this study, a novel ArdK binding sequence (CUPA56) was identified upstream of *bla*_IMP-6_ on pA56-1S, suggesting that the expression of *bla*_IMP-6_ is repressed by the binding of ArdK to CUPA56 in A56-1S (Fig 4). The consensus sequence of CUPA56 (4,812–4,824 nt in pA56-1S) comprises 10 nucleotides of *dfrA* and 3 nucleotides of the inverted repeat of IS*26*, suggesting that CUPA56 is the result of the insertion of IS*26* into *dfrA*.

The *ardK* gene disruption was caused by the insertion of duplicated IS*26* into the *ardK* gene in A56-1R. Moreover, IS*26* plays a major role in the acquisition and dissemination of antibiotic resistance in gram-negative bacteria [20–23], and many bacteria that have developed antibacterial resistance as a result of IS*26*-related transposition have been isolated from hospitals [24,25].

The results of the EMSA and biolayer interferometry assay indicate that ArdK can act as a transcriptional regulator, with a *K*d value comparable to the binding affinity of well-characterized regulators such as RNA polymerase-σ^54^ and SoxS (Fig 4) [26–28].

The pA56-1S plasmid was shown to be similar to the pKPI-6 IncN plasmid carrying *bla*_IMP-6_ and *ardK* in the *K*. *pneumoniae* KPI-6 strain, which was isolated in Japan in 2009 [9], suggesting that these plasmids could have the same origin (Fig 2). Enterobacteriaceae carrying pKPI-6-related plasmids have also been identified in various regions of western Japan [17]. The sequence upstream of *bla*_IMP-6_ on pKPI-6 was distinct from that on pA56-1S (Fig 2), and the ArdK binding sequence CUPA56 was not found on pKPI-6, suggesting that ArdK could not downregulate *bla*_IMP-6_ expression on pKPI-6 because of a lack of CUP elements.

IMP-6 producers such as KPI-6 and A56-1R exhibit an ISMR phenotype because the carbapenemase activity of IMP-6 MBL against IPM is far weaker than that against MEM [9,10], and are difficult to identify as CPE based on IPM susceptibility alone [9,17]. However, when using the high sensitivity Carb NP test, IMP-6 producers showing the ISMR phenotype were detectable (Fig 1) [7]. A56-1S, which exhibits an ISMS phenotype (the MEM MIC of A56-1S is below the screening cut-off value) due to the low expression of IMP-6 MBL, is undetectable by antimicrobial susceptibility testing and even by the Carba NP test. Therefore, genetic screening methods, such as PCR and whole-genome sequencing, are indispensable for detecting ISMS phenotype strains and preventing the dissemination of these strains in hospitals and community environments.

In conclusion, comparative analysis between IMP-6 MBL non-producing A56-1S and producing A56-1R strains revealed that the transcription factor ArdK contributes to repression of *bla*_IMP-6_ transcription (Fig 5). The mechanism of repression by ArdK was analyzed with a reporter assay, EMSA, and kinetic characterization. In these results, it was demonstrated that ArdK plays a key role in the repression of IMP-6 MBL production by binding to the regions upstream of *bla*_IMP-6_ (CUP elements), leading to the ISMS phenotype of A56-1S. Most bacteria that exhibit an ISMS phenotype might be undetectable via routine antimicrobial susceptibility tests, and these bacteria might contribute to the dissemination of carbapenemase genes. Consequently, the ISMS phenotype bacteria play a potential role as undetected reservoirs of the carbapenemase gene.
